# Comparative analysis of ROCKET-driven and classic EEG features in predicting attachment styles

**DOI:** 10.1186/s40359-024-01576-1

**Published:** 2024-02-22

**Authors:** Dor Mizrahi, Ilan Laufer, Inon Zuckerman

**Affiliations:** Department of Industrial Engineering and Management, Ariel University, Ariel, Israel

**Keywords:** EEG (Electroencephalogram) features, ROCKET algorithm (RandOm Convolutional KErnel transform), Machine learning in EEG Data Analysis, Neural Signal Analysis, Attachment styles

## Abstract

Predicting attachment styles using AI algorithms remains relatively unexplored in scientific literature. This study addresses this gap by employing EEG data to evaluate the effectiveness of ROCKET-driven features versus classic features, both analyzed using the XGBoost machine learning algorithm, for classifying ‘secure’ or ‘insecure’ attachment styles.

Participants, fourth-year engineering students aged 20–35, first completed the ECR-R questionnaire. A subset then underwent EEG sessions while performing the Arrow Flanker Task, receiving success or failure feedback for each trial.

Our findings reveal the effectiveness of both feature sets. The dataset with ROCKET-derived features demonstrated an 88.41% True Positive Rate (TPR) in classifying ‘insecure’ attachment styles, compared to the classic features dataset, which achieved a notable TPR as well. Visual representations further support ROCKET-derived features’ proficiency in identifying insecure attachment tendencies, while the classic features exhibited limitations in classification accuracy. Although the ROCKET-derived features exhibited higher TPR, the classic features also presented a substantial predictive ability.

In conclusion, this study advances the integration of AI in psychological assessments, emphasizing the significance of feature selection for specific datasets and applications. While both feature sets effectively classified EEG-based attachment styles, the ROCKET-derived features demonstrated a superior performance across multiple metrics, making them the preferred choice for this study.

**Supplementary Information:**

The online version contains supplementary material available at 10.1186/s40359-024-01576-1.

## Introduction

In the intersection of developmental psychology and neuroscience, attachment theory posits that early-life interactions with primary caregivers shape distinct attachment styles, categorized as ‘secure’ or ‘insecure’ [1, 2]. These attachment styles exert substantial influence on adult relationships, emotional responses, and coping strategies, making their accurate characterization crucial for therapeutic and developmental interventions.

Electroencephalogram (EEG) is a powerful tool for uncovering the neural foundations of psychological constructs such as attachment styles [3–8]. Researchers have used EEG signals to extract various features like frequency attributes, complexity metrics, and time-domain characteristics, allowing a thorough examination of the neural basis of attachment styles (e.g. [3–5]). While AI models and EEG data are extensively used for emotion prediction [9–13], the specific application of AI models to EEG data for studying attachment styles remains unexplored, despite the use of techniques based on vocal and conversational characteristics [14, 15].

To enhance our predictive capabilities in the domain of attachment styles, we explore cutting-edge machine learning algorithms. XGBoost, a robust gradient boosting algorithm [16], shows promise for predicting both secure and insecure attachment styles from EEG data. XGBoost’s structure, which inherently emphasizes feature importance, flexibility in hyperparameter tuning, and adeptness at handling structured data, makes it a contender worth considering for EEG datasets [17].

In this study, we assess its performance using two distinct feature sets. The first set comprises 45 classic EEG features (for more details see Appendix A), including time-domain features [18, 19], complexity features [20], frequency based features [21], and task-related features. The second set is derived from the ROCKET (RandOm Convolutional KErnel Transform) algorithm [22], where 20,000 features are extracted and subsequently reduced to 87 principal components using PCA. This comparative analysis within the XGBoost framework will advance our understanding of attachment style prediction in the context of neural signals. This approach allows a controlled comparison, isolating the impact of the feature sets on predictive performance. Utilizing the robustness and versatility of XGBoost ensures that differences in results can be attributed to the features themselves, providing clear insights into the effectiveness of classical EEG features versus features derived from the ROCKET algorithm and PCA.

The ROCKET algorithm plays a pivotal role in time series classification [22]. It employs a wide array of randomly selected convolutional kernels to transform time series data into interpretable features, revealing significant patterns from time series data, regardless of its size or complexity. However, ROCKET predominantly serves as a feature extractor and is not directly used for classification in our study. Conversely, XGBoost, known for its architectural robustness [23, 24], has an ensemble technique that pools insights from multiple decision trees, adeptly addressing noise and variability inherent in EEG datasets [25]. This makes it a suitable model for comparing the effectiveness of different feature sets in predicting attachment styles.

In summary, our study evaluates the performance of XGBoost in predicting ‘secure’ and ‘insecure’ attachment styles using two different sets of features. One set is derived from classical EEG analysis, and the other is based on the advanced feature extraction capabilities of the ROCKET algorithm, followed by PCA for dimensionality reduction. The objective is to determine which feature set offers more reliable results when used with the XGBoost model, providing insights for potential future research and applications in neuroscience and psychology.

## Methods

### Participant selection and EEG data acquisition procedures

The study explored attachment styles using a two-step approach. In the first phase, 96 participants, primarily fourth-year engineering students aged between 20 and 35 years (average age = 24.25, SD = 2.0673), were selected. Our selection of the 20–35 year age range aligns with the developmental dynamics of young adulthood as discussed in existing literature [26, 27]. These cited studies, while not explicitly addressing feedback sensitivity, highlight the emotional and psychological changes characteristic of this life stage. Considering that our study focuses on fourth-year students, who are typically within this age range and experiencing the transitional phase from university to professional life, it is reasonable to infer that these changes could influence sensitivity to emotional feedback that we explore in our study through feedback responses in cognitive tasks. The wide age range serves as an advantage, allowing us to explore attachment styles and feedback responses across a broader spectrum of life experiences within young adulthood.

It was ensured that all participants were right-handed and did not exhibit any neurological symptoms. We chose to include only right-handed participants due to documented differences in brain hemisphere dominance between right- and left-handed individuals [e.g [28]]. Given our heavy reliance on EEG data, which can be influenced by neural variations, this decision ensured consistency and minimized potential EEG signal confounders. The attachment styles of participants were assessed using the [29, 30] questionnaire. Subsequently, participants were categorized into four distinct attachment categories. The k-means algorithm [31] (k = 4) was utilized for this classification, resulting in a secure group comprising 6 individuals and an insecure group encompassing 21 participants (9 anxiously attached, 7 avoidant, 5 fearful avoidant). From this initial cohort, 27 individuals were meticulously chosen for the EEG sessions of the second phase, ensuring representation from both the secure and insecure attachment groups.

The selection process for these 27 participants was informed by the principles of proportional allocation, a method designed to ensure a sample representative of the initially assessed attachment styles. This method involves selecting individuals in such a way that the sample contains the same proportions of each subgroup as observed in the larger initial population. By employing this technique, we guaranteed that our smaller sample of 27 participants maintained the diversity of attachment styles present in the initial cohort. This selection was executed randomly within each attachment style subgroup, providing an unbiased representation of the population.

**Fig. 1:**
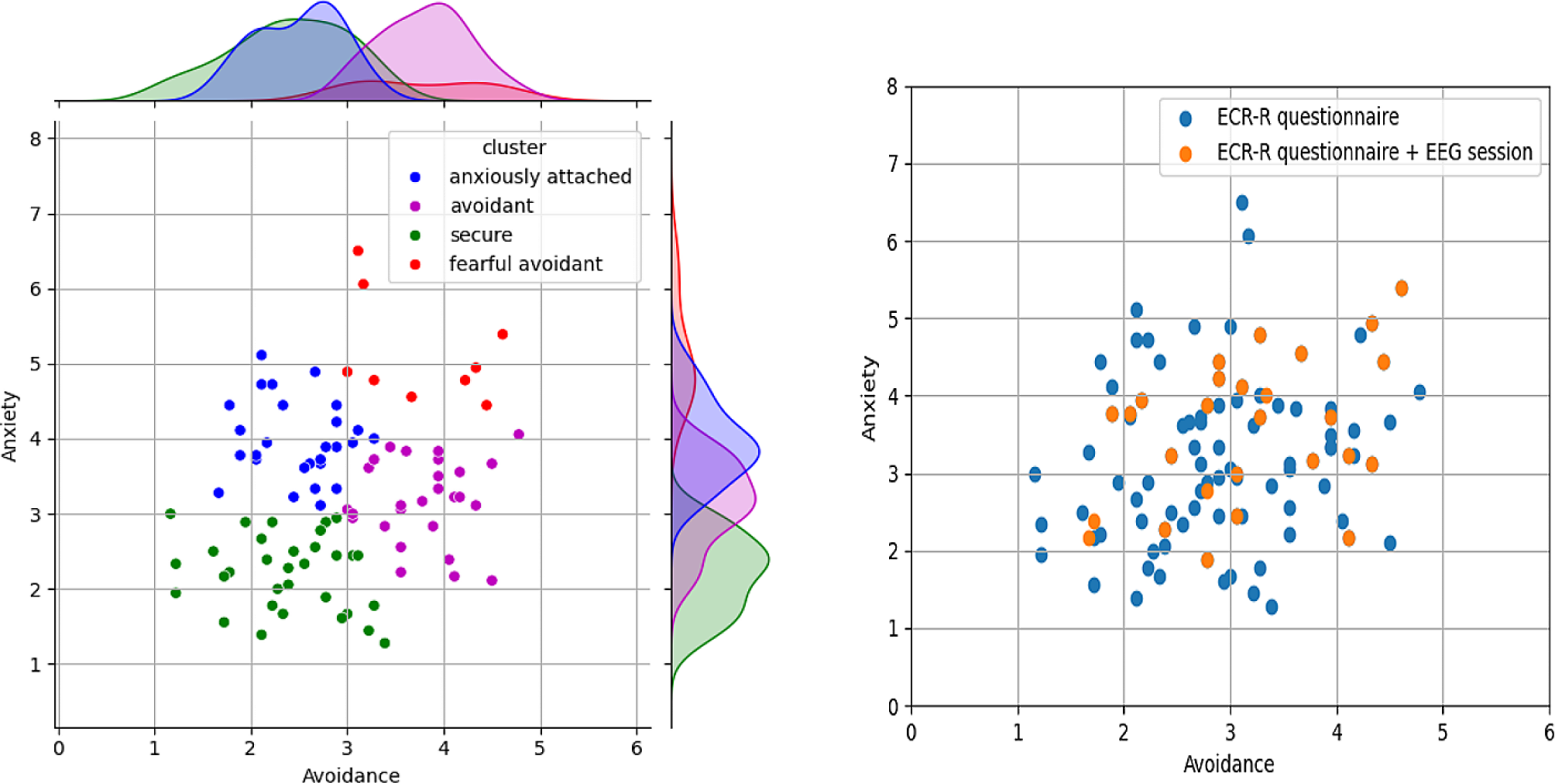
**(A)** Distribution of attachment styles in the initial cohort of 96 participants, as classified by the ECR-R questionnaire. **(B)** Subgroup of 27 participants selected for the EEG session, illustrating the proportional representation of various at

Figure 1A and B visually depict the distribution of attachment styles within our study’s population. Figure 1A illustrates the distribution of attachment styles as measured by the ECR-R questionnaire for all participants. Figure 1B, in contrast, highlights the 27 participants who progressed to the EEG session, shown against the backdrop of the initial cohort from Fig. 1A. These participants are denoted as “ECR-R questionnaire + EEG session” in the legend (designated in orange). Figure 1B vividly demonstrates the heterogeneity of our EEG participant sample, showcasing a broad scatter across the attachment style spectrum. This reinforces the randomness and representativeness of our selection method, affirming that we have succeeded in capturing a wide-ranging and unbiased snapshot of attachment styles in our study.

**Fig. 2:**
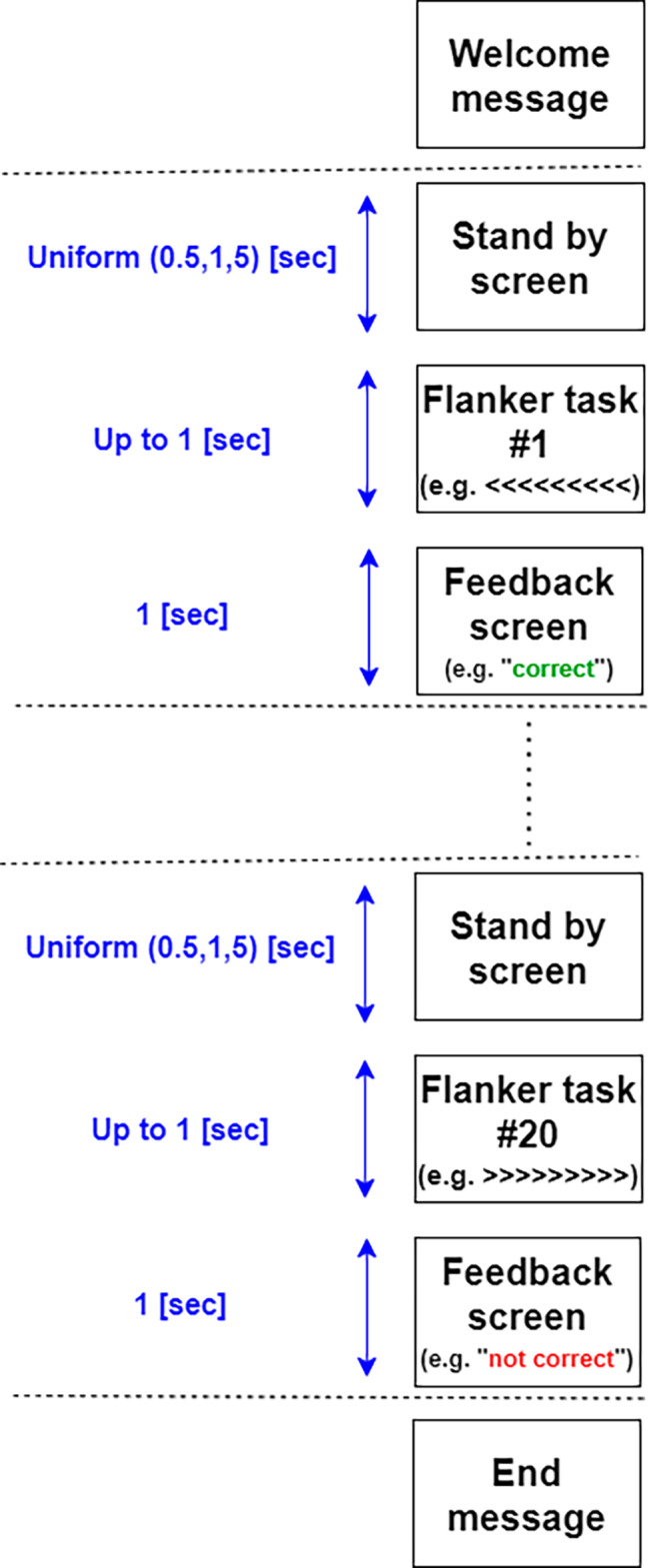
Experimental paradigm– single block

To make the analysis more efficient, the attachment styles were consolidated into two broad categories: secure and insecure. The emphasis was on pinpointing differences between these groups. In the EEG sessions, participants were exposed to the flanker task [32], which was divided into 60 trials. These trials were further subdivided into three blocks, each containing 20 trials (see Fig. 2), with a break of one minute in between. Participants were asked to respond to arrows on a screen. In the first and third blocks, they responded in the direction of the arrow, while in the second block, they chose the opposite direction. After each trial, they received feedback in the form of a green or red indicator, signifying “correct” or “incorrect” respectively, which was displayed for one second. In between trials, participants’ focus was directed to a gray cross on a black background, shown anywhere between 0.5 and 1.5 s. Each trial lasted about 3 s, making each 20-trial block roughly 60 s. A preliminary session was conducted to acquaint participants with the task.

For the acquisition of EEG data, a 16-channel EEG system (USBAMP by g.tec, Austria) was used. It had a sampling rate of 512 Hz and adhered to the 10–20 international system. The focus was on six frontal and prefrontal electrodes: Fp1, F7, Fp2, F8, F3, and F7. The recorded EEG data underwent a bandpass filter ranging between 1 and 30 Hz to minimize noise interference. Subsequently, the Independent Component Analysis (ICA) was employed to distinguish neural signals from any potential artifacts. A total of 1600 epochs were collected, with 20 epochs excluded from the dataset due to poor-quality recordings from one participant. The data was then partitioned into 1-second epochs, synchronized with the timings of the flanker task slide.

### Classification

The aim of our analysis was to utilize EEG data for classifying individuals as ‘secure’ or ‘insecure’. We employed a dual-feature set approach in our analysis. Initially, a comprehensive set of 45 features was extracted from the EEG epochs, distributed across the following categories:

- Frequency-Based Features: These encompassed 6 attributes related to Alpha, Beta, Theta, and Delta frequency bands, along with the Theta to Alpha and Theta to Beta ratios.
- Complexity-Based Features: This group comprises 17 features designed to measure the complexity of EEG signals. Key features encompass Fourier entropy (evaluated across diverse bin sizes such as 2, 4, 8, 16, and 32), Lempel-Ziv Complexity (LZC) (analyzed across comparable bins), Complexity-Invariant Distance Measure (CID_CE), and the mean of sample entropy.
- Time Domain-Based Features: This category includes 19 attributes. Examples include the average of absolute energy values, the mean of maximum absolute values, the average measure of data distribution tails (kurtosis), the mean measure of data asymmetry (skewness), and the mean of relative variability.
- Trial Feedback Dynamics: This category encompasses 3 features, specifically capturing the response time and the valence of success/failure feedback from both the current trial and the preceding one. These features are crucial facets of the feedback mechanism.

Our inclusion of trial-feedback dynamics was primarily based on the expectation that these dynamics would significantly affect participants’ emotional coping mechanisms. Given the strong link between emotional responses and EEG activity, we anticipated that these emotional coping responses to feedback would, in turn, influence the EEG frequency patterns. This approach aimed to elucidate how variations in emotional coping driven by feedback types could manifest as distinct electrophysiological patterns, particularly in the context of different attachment styles. The selection of a decision tree-based classification model was strategic for this study. Its ability to process binary variables, such as success/failure feedback, enhances the interpretation of EEG data. This model effectively identifies how emotional responses to feedback, linked to different attachment styles, influence EEG patterns, thereby deepening our insight into the neural basis of attachment-related emotional reactions.

For a detailed breakdown of the 45 classic EEG-based features utilized in this research, please refer to Table A1 in Appendix A. Additionally, we explored the utility of ROCKET [22, 33] a machine-learning algorithm renowned for its rapid and efficient feature extraction from time series data. Using ROCKET, we generated an extensive feature set of 20,000 attributes. To manage the complexity, we implemented Principal Component Analysis (PCA), effectively distilling this down to 87 principal components for our classification needs.

For classification, both the 45 EEG-specific features and the 87 ROCKET-derived principal components were separately analyzed using the XGBoost algorithm [23, 24]. The dataset, comprising 1600 epochs, was partitioned into four folds for k-fold cross-validation. In each iteration, 1280 epochs were allocated for training and 320 epochs for testing. This systematic approach ensured a comprehensive evaluation using varied subsets of data, thereby aiming to bolster the model’s generalization capability. Post-classification, we computed confusion matrices for both models to gauge their prediction accuracy.

To summarize, two distinct feature sets were analyzed using the XGBoost algorithm: one comprising 45 EEG-specific features and the other consisting of 87 principal components derived from ROCKET. Both these models were instrumental in differentiating between “secure” and “insecure” individuals based on their EEG data.

### Model performance across the attachment style continuum

Attachment styles are not simply categorical but exist along a continuum. This continuum is mapped on a two-dimensional plane, with its axes representing anxiety and avoidance, two pivotal constructs of attachment styles. The two-dimensional plane’s origin serves as a reference point, representing a balanced presence of both anxiety and avoidance. We introduced a metric, $$ {R}_{attachment}$$, defined by the equation:

1 $$ $${R_{attachment}} = \sqrt {{{\left( {avoidance} \right)}^2} + {{\left( {anxiety} \right)}^2}} $$ $$

This metric quantifies the distance of an individual from the origin and thereby offers insight into their position on the attachment style spectrum.

**Fig. 3:**
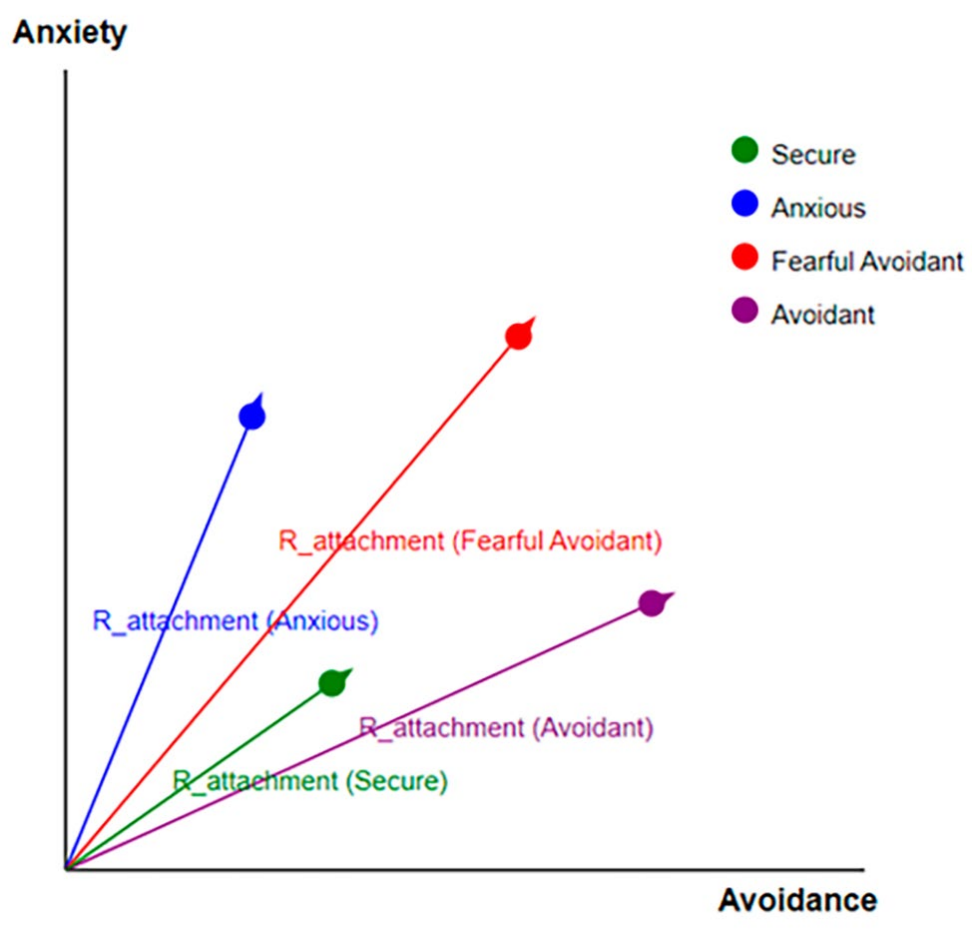
A schematic representation of attachment styles plotted on “Avoidance” and “Anxiety” axes with radii indicating intensity

Figure 3 displays a quadrant, showcasing the spread of four distinct attachment styles in relation to “Avoidance” and “Anxiety,” the primary dimensions derived from the ECR-R questionnaire. Secure attachment, near the origin, indicates balanced avoidance and anxiety levels. Anxious individuals display higher anxiety and moderate avoidance, while fearful-avoidant individuals exhibit heightened values for both dimensions. Avoidant individuals are characterized by high avoidance levels.

Radii in Fig. 3, denoted as R_attachment, extend from the origin to each dot, quantifying the deviation of each style from a neutral midpoint. They highlight the intensity within the “Avoidance” and “Anxiety” dimensions. Longer radii suggest more accentuated traits, and shorter ones indicate a balanced approach. The radii provide both a quantitative metric for comparison and intuitive insights into the predominant dimension guiding each style. The interpretation of these radii varies: longer radii indicate higher intensity for insecure tendencies, while a shorter radius for secure styles indicates strong secure attachment behaviors or feelings.

## Results

This section presents the outcomes of our analysis, which evaluated the classification performance of XGBoost in predicting attachment style class as either ‘secure’ or ‘insecure’ based on EEG data. The key distinction lies in the comparison of two feature sets used for prediction: one comprising 45 classic EEG features, and the other utilizing 87 features derived from the ROCKET algorithm and PCA. For the PCA applied in the ROCKET feature extraction process, we adopted a criterion where the principal components were selected to retain over 90% of the variance, ensuring a comprehensive representation of the data with reduced dimensionality.

Table 1 encapsulates key metrics derived from confusion matrices to evaluate the effectiveness of the predictions using these two feature sets.

**Table 1:** Comparison of prediction performance: classic EEG features vs. ROCKET-derived features

Metrics	Classic Features	ROCKET Features
True Positive (Insecure)	981	1114
False Negative (Insecure)	259	126
False Positive (Secure)	39	34
True Negative (Secure	321	326
True Positive Rate	79.11%	88.41%
True Negative Rate	89.17%	90.56%
Positive Predicted Value	96.18%	97.04%
False Discovery Rate	3.82%	2.96%
Prediction Accuracy	81.37%	87.5%

For the classification of the ‘insecure’ attachment style, the ROCKET-derived features exhibit a clear advantage. A True Positive (TP) count reflects the number of ‘insecure’ individuals correctly identified: 1,114 for ROCKET-derived features compared to 981 for classic EEG features. The false negative count, representing ‘insecure’ individuals mistakenly classified as ‘secure’, was notably lower for ROCKET-derived features at 126, contrasting with classic EEG features’ 259.

The significant improvement in the True Positive Rate for ROCKET-derived features (88.41% versus 79.11%) holds substantial practical implications. This elevated rate suggests that ROCKET-derived features are superior in detecting actual insecure cases. Additionally, both ROCKET-derived features and classic EEG features exhibit high Positive Predictive Values (PPV) for the ‘Secure’ class. In practical terms, this means that both feature sets have a strong ability to correctly classify situations as ‘secure’ when they are indeed ‘secure.’ However, it’s important to note that ROCKET-derived features’ slightly enhanced PPV, although not substantially different from classic EEG features’, still contributes to greater confidence in its classifications. This confidence can be especially valuable in scenarios where trust in predictions is critical. In essence, the elevated True Positive Rate (TPR) ensures that fewer insecure cases remain unnoticed, while the amplified PPV offers greater confidence when the feature set designates a case as ‘secure’, especially in scenarios where faith in such a prediction is paramount.

For the ‘secure’ attachment style, the results were more closely matched between the two feature sets. The True Negative Rate (TNR) (indicating ‘secure’ individuals correctly identified) stood at 326 for ROCKET-derived features and 321 for classic EEG features. A minor difference was observed in false positives, with ROCKET-derived features registering 34, slightly better than classic EEG features’ 39. Further examination of the rates and predictive values continues to favor ROCKET-derived features. The True Negative Rate, indicating the proportion of correctly identified ‘secure’ cases, was 90.56% for ROCKET-derived features, compared to 89.17% for classic EEG features. The False Discovery Rate (FDR), which calculates the probability of falsely categorizing an individual as ‘insecure’, was lower for ROCKET-derived features at 2.96%, compared to classic EEG features’ 3.82%. Reducing the FDR is particularly important in scenarios where the consequences of misclassification are significant. For example, in applications related to mental health, misclassifying a secure individual as insecure could lead to unnecessary interventions or distress. Therefore, a lower FDR contributes to the reliability and trustworthiness of the model’s predictions.

In terms of overall prediction accuracy, representing the total accurate classifications, ROCKET-derived features demonstrated superiority with 87.5%, compared to classic EEG features’ 81.37%. In conclusion, while both feature sets proved effective in EEG-based attachment style classification, ROCKET-derived features displayed superior performance across various metrics, rendering it the preferred choice for this application.

**Fig. 4:**
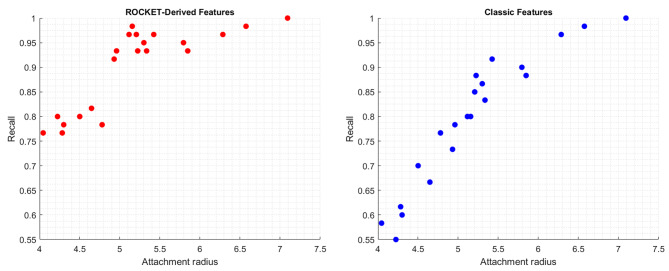
Attachment radii vs. recall values for two feature sets. Panel A (left) presents recall values using ROCKET-derived features, while Panel B depicts those from classic EEG features. The emphasis is on the true positive rate (TPR) improvement for the ins

Displayed below are the visual representations of two predictive models’ performance in relation to attachment radii and recall values for the insecure group (Fig. 4A and B). In Panel A (left), the relationship is depicted using ROCKET-derived features, while Panel B portrays the same using classic EEG features. The emphasis on the insecure group’s improvement is particularly pertinent due to the critical role of the True Positive Rate (TPR) in our analysis. A high TPR is essential as it indicates the model’s proficiency in correctly identifying individuals with insecure attachment traits. The observed significant rise from 79.11% using classic EEG features to 88.41% with ROCKET-derived features underscores the significance of optimizing prediction models to accurately and reliably pinpoint participants exhibiting insecure attachment behaviors.

For the use of ROCKET-derived features (Fig. 4A), there’s a noticeable distinction in data point density between the attachment radii of approximately 4 to 5. Within this range, there are fewer participants, which might suggest potential outliers or specific behavioral traits less prevalent in the population. However, despite the reduced density, the recall values within this segment are mainly between 0.75 and 0.8. Beyond this range, particularly between the radii of approximately 4.5 to 7.5, participants exhibit recall values primarily between 0.9 and 1, reflecting the consistent proficiency in predicting insecure attachment behaviors when utilizing ROCKET-derived features.

In contrast, the figure for the prediction using classic EEG features (Fig. 4B) illustrates a broader distribution in recall values, spanning from about 0.55 to 0.95. An upward trend in recall values is apparent as the attachment radii increase, emphasizing the model’s improved accuracy for participants with increasing attachment radii. This behavior suggests that the model using classic EEG features is adept at identifying pronounced insecure tendencies, but there could be challenges at the lower radii, where the boundary between secure and insecure traits is more nuanced, potentially leading to misclassifications.

In conclusion, the use of ROCKET-derived features showcases high recall values across most attachment radii but does present a distinct gap in data point density between the radii of 4 to 5. This section might benefit from more detailed research or data gathering. The model using classic EEG features, though showing a trend of enhanced recall values with larger attachment radii, might face challenges at lower radii underscoring its potential difficulty in discerning the nuanced boundary between secure and insecure individuals.

**Fig. 5:**
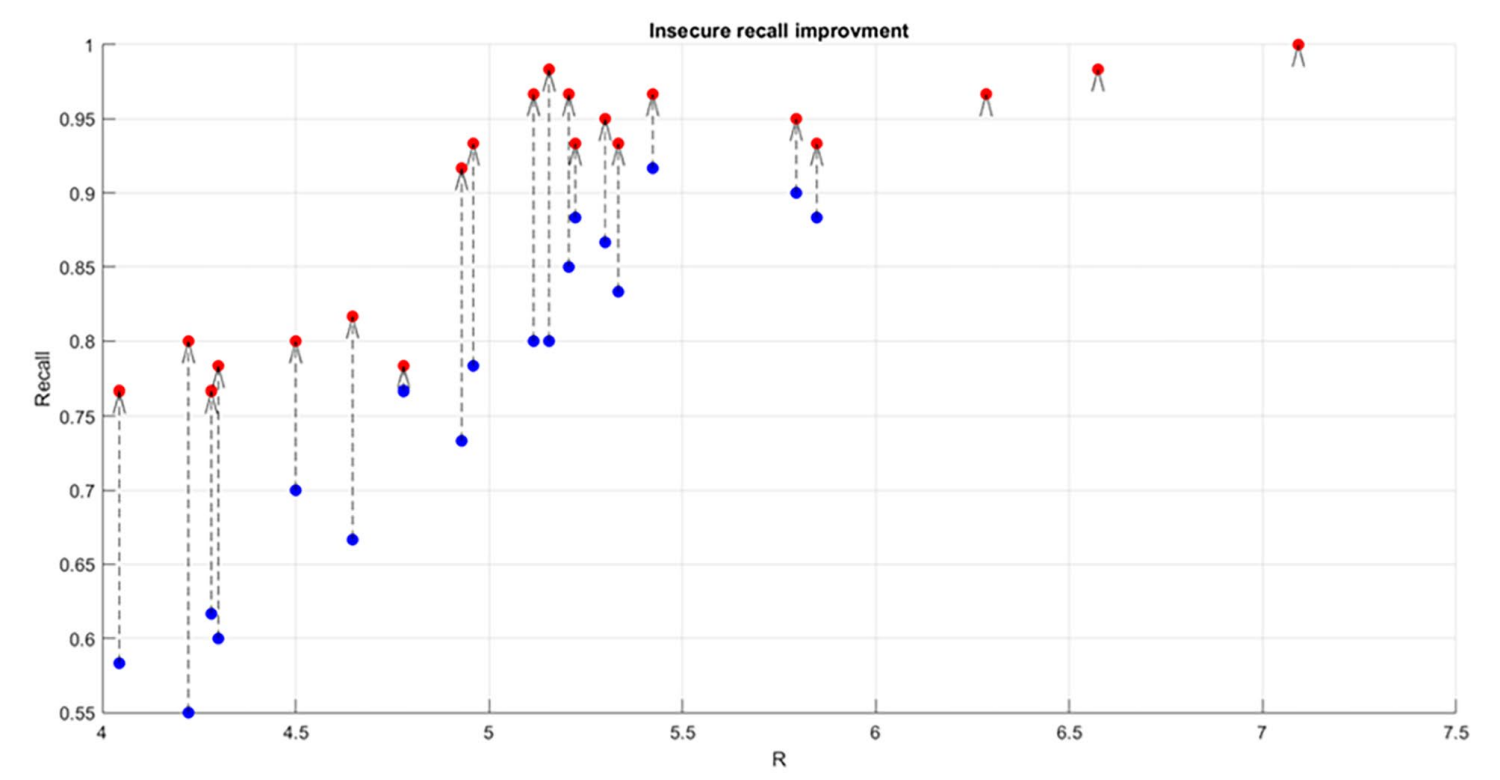
Paired comparison of recall values for insecure participants: classic EEG features (blue dots) vs. ROCKET-derived features (red dots) across different attachment radii

Figure 5 displays the comparative recall performance for the insecure group using two feature sets: classic EEG features (blue dots) and ROCKET-derived features (red dots). Dashed lines connect pairs of dots representing individual participants’ recall values with each feature set. The most notable improvement is observed in the 4.5 to 5.5 attachment radii range, where ROCKET-derived features significantly enhance recall values, as seen by the consistently higher red dots. This enhancement is particularly distinct for participants who initially had lower recall values with classic EEG features, as indicated by the substantial elevation of their red dots, signifying an overall notable improvement with ROCKET-derived features. While the improvement is less marked beyond the 5.5 radius, ROCKET-derived features still maintain a performance edge at some radii.

## Discussion

In our study, we aimed to evaluate the effectiveness of two distinct datasets in predicting attachment styles, specifically ‘secure’ and ‘insecure,’ using EEG data. The first dataset comprised classic EEG features, while the second utilized features derived from the ROCKET [22, 33] transformation algorithm. This approach contributes to the field of time series classification and sheds light on the impact of feature extraction methods in the context of EEG data analysis.

### Comparative analysis

Our analysis revealed a notable preference for ROCKET-derived features in classifying attachment styles, particularly for distinguishing the ‘insecure’ attachment style. When utilizing classic EEG features, the True Positive Rate (TPR) was moderately effective. However, with the introduction of ROCKET-derived features, there was a significant increase in TPR, indicating a marked improvement in identifying individuals with ‘insecure’ attachment traits. This difference underscores the importance of feature extraction and transformation methods in dealing with complex EEG datasets.

The enhanced performance of ROCKET-derived features is due to the algorithm’s specific design for processing time series data, which is fundamental to EEG recordings. ROCKET’s capacity to identify patterns within the EEG data with its array of random convolutional kernels was crucial. Meanwhile, models using classic EEG features can be effective, but they might encounter difficulties in handling the nuanced and time-sensitive characteristics present in EEG data.

#### The $$ {R}_{attachment}$$ metric

We introduced the $$ {R}_{attachment}$$ metric to provide a nuanced understanding of attachment styles as a continuous spectrum. When analyzed using classic EEG features, there was variability in predictive accuracy, particularly for individuals with milder attachment behaviors. However, with the introduction of ROCKET-derived features, there was a consistent high accuracy across a range of attachment radii, indicating its proficiency in identifying individuals with pronounced insecure tendencies. This finding supports the use of advanced feature extraction methods like ROCKET in enhancing predictive performance. Our study aligns with the discoveries of [34]. Their evaluation of various algorithms for multivariate time series classification (MTSC) on 26 equally sized problems from the UEA MTSC archive further elucidates the strengths of ROCKET [34]. found that ROCKET not only outperforms the robust benchmark of dynamic time warping (DTW) but also emerges as the preferred choice for MTSC problems due to its exceptional accuracy and remarkably swift training times. This concurs with our own observations regarding ROCKET’s adaptability across various $$ {R}_{attachment}$$ Radii values, showcasing its capacity to effectively capture intricate data patterns.

### Research context and comparative insights

In our investigation, we explored the potential of ROCKET-derived features in EEG data analysis and compared their performance against a dataset utilizing 45 classic features. This comparative approach underscores the significance of feature selection in EEG data analysis. Our findings resonate with the broader research landscape, which positions ROCKET as a potent tool in time series classification due to its ability to generate a diverse array of features efficiently [22, 33]. The versatility of ROCKET, further augmented by extensions like MiniROCKET [35] and MultiROCKET [36], reaffirms its suitability for EEG data analysis. However, our study also emphasizes that the choice of features can significantly impact the model’s performance, a nuance that is often overlooked.

Contrasting findings in the field further highlight the complexity of EEG data analysis. For instance, [37] examined the multi-scale ROCKET approach and found a disparity in performance when compared to a specialized multi-feature SVM model in detecting inter-bursts in preterm EEG. This echoes our observation that the efficacy of a model can vary depending on the features used and the specific task at hand. Such nuances necessitate a tailored approach in algorithm and feature selection.

 [38] extended the discussion by comparing the performance of unsupervised learning methods, including ROCKET and XGBoost, for seizure identification in EEG data. Although both models demonstrated commendable performance, the slight edge of ROCKET over XGBoost in some instances, and vice versa, underscores that no single model or feature set is universally superior. This finding aligns with our study, which showcases the importance of considering both advanced feature extraction methods and traditional features based on the specific dataset and task.

 [39] further illuminated the variability of model performance based on dataset characteristics, revealing instances where classic tabular models outperformed advanced time series models like ROCKET. This insight is particularly relevant to our study, as it reinforces the notion that the characteristics of the dataset can significantly influence the effectiveness of the chosen features and models.

In summary, while our study reinforces the efficacy of advanced feature extraction methods like ROCKET in EEG data analysis, the insights gathered from our comparative analysis and the broader research context guide us in achieving a balance between embracing state-of-the-art models and recognizing the potential of simpler approaches, depending on the task at hand.

### Practical recommendations for EEG data analysis: balancing advanced and classic approaches

Our study validates the efficacy of advanced feature extraction methods like ROCKET in EEG data analysis, while also highlighting the potential of classic feature sets. Based on our comparative analysis and the broader research context, we propose the following practical recommendations:

1. **Dataset-Specific Feature Selection**: Conduct preliminary analysis to understand your EEG dataset’s unique characteristics, guiding the choice between advanced features like ROCKET-derived and classic ones.
2. **Hybrid Feature Approach**: For complex or heterogeneous datasets, consider combining classic and advanced features to achieve a more comprehensive data representation.
3. **Model Flexibility**: Test multiple models. Advanced models like XGBoost and ROCKET are powerful, but in some cases, simpler models may be more effective.
4. **Performance Benchmarking**: Use simpler models and features as benchmarks to evaluate the added value of more advanced methods.
5. **Customized Feature Engineering**: Analyse the specific attributes and anomalies present in your EEG data. Develop custom features that directly address these unique aspects, such as specific waveforms, frequency bands, or patterns linked to the condition under study. Employ domain knowledge and data exploration to create features that are finely tuned to the nuances of your dataset.
6. **Cross-Dataset Validation**: To ensure robustness and generalizability, validate your model and feature selection across different datasets whenever possible.

In light of our findings, coupled with the insights gleaned from the existing scientific literature, we recommend that researchers and practitioners critically evaluate their approach to EEG data analysis. While the ROCKET-driven features stand out for their adaptability to the dynamic EEG data, classic features remain an invaluable resource due to their interpretability and longstanding use in the field. Depending on the study’s goals and the nature of the dataset, one might be favoured over the other.

### Limitations and future studies

Our study provides insights into the use of machine learning for predicting attachment styles from EEG data, using two distinct datasets: one with 45-classic features and another with ROCKET-derived features. Despite the promising findings, it’s important to acknowledge several limitations and identify areas for further research:

1. **Participant Demographics**: Our sample primarily consisted of students, limiting the generalizability of our findings to a broader population. Moreover, the proportional allocation method used in participant selection resulted in lower representation of ‘secure’ attachment styles, potentially impacting the applicability of our results.
2. **Feature Extraction and Comparison**: While we explored spectral, complexity, and temporal metrics from EEG data, there are numerous unexplored features. Future research should delve into additional metrics such as cross-frequency coupling or phase-amplitude coupling to compare their predictive power across the two datasets.
3. **Consideration of Feature Set Size**: In evaluating the performance of ROCKET-driven features against classic features in our analysis, we acknowledge a notable difference in the number of features used—87 for ROCKET and 45 for classic features. This discrepancy might raise concerns about the basis for comparison. However, it’s essential to consider the intrinsic nature of these feature sets. The ROCKET-derived features, by design, generate a large number of features to capture the intricate dynamics of EEG data. In contrast, classic features are fewer but are selected based on their established relevance and interpretability in EEG analysis.

The comparison, therefore, is not solely based on the quantity of features but on their qualitative aspects and the predictive insights they offer. Our aim was to evaluate whether the additional complexity and volume of features generated by ROCKET translate into a correspondingly significant improvement in predictive performance. The analysis using XGBoost, a consistent algorithm for both sets, provided a common ground for this evaluation. While there is an inherent difference in the feature sets, the consistent methodology in their application and analysis offers a basis for comparison, allowing us to assess the trade-offs between feature complexity, quantity, and predictive efficacy. Future studies might consider standardizing the number of features or employing feature selection techniques to further refine this comparison.

4. **Delta Frequency Resolution and Window Selection**: The extraction of classical features from one-second EEG trials, including those related to the delta frequency band (1–4 Hz), may raise questions about the adequacy of the chosen time segment for reliably estimating low-frequency components. However, it’s important to note that the one-second duration was selected to align with the minimum frequency resolution of the delta band (1 Hz) based on our sampling frequency of 512 Hz. This choice was made to ensure that our analysis accurately captured the characteristics of the delta frequency and is also in line with similar methodologies reported in the literature [40]. Furthermore, the one-second window reflects the activity immediately following the trigger of the feedback slide, which corresponds to success/failure feedback. While extending the window might provide additional data, it could potentially deviate from our specific focus on success/failure feedback, which is central to our EEG segment analysis. Future studies might explore extended window durations, assessing their impact on delta frequency estimation while considering the trade-off between temporal specificity and data comprehensiveness.
5. **Addressing Feature Heterogeneity in EEG Classification**: Our study’s use of XGBoost is supported by its demonstrated proficiency in handling feature heterogeneity. For example, it was shown that tree-based methods like XGBoost are effective for high-dimensional data, implicitly addressing heterogeneity by selecting the most relevant features for analysis [41]. Additionally, another study demonstrates XGBoost’s superior performance in environments with diverse data types, further validating its capability in managing heterogeneous datasets [42]. These studies affirm our rationale for employing XGBoost, highlighting its strength in feature selection and adaptability to varied data characteristics.

However, it is important to acknowledge potential limitations and future research directions. there are potential areas for enhancement. Advanced feature selection as highlighted in research [43] could augment XGBoost’s capabilities. The former research introduces a hybrid feature selection method for EEG data that combines Maximum Information Coefficient (MIC) and Quantum Particle Swarm Optimization (QPSO). MIC is used for eliminating irrelevant and redundant features, effectively reducing the search space. QPSO is then employed to optimize the feature set in the second stage, aiming for an optimal feature subset with high classification accuracy and low computational complexity. These methods can streamline feature dimensionality while retaining crucial information, potentially boosting classification accuracy. integrating such methods with XGBoost could further enhance classification accuracy. Exploring these advanced techniques represents a promising direction for future research in EEG data analysis, especially in complex neuroscientific and psychological contexts.

6. **Research avenues for Improved comparison:**

- **Exploring Diverse Features**: Investigating how various features perform across the two datasets could provide deeper insights. Comparing the performance of complex network-based features (e.g., Degree Centrality, Clustering Coefficient) or physiological indicators like heart-rate variability in both datasets might reveal crucial differences in how these features correlate with the outcome or predict the trait of interest.
- **Temporal Analysis**: Examining neural patterns over different time scales could reveal how attachment styles manifest differently across datasets. This extended temporal analysis could offer comparative insights into neural dynamics.
- **Data Diversity and Cross-Dataset Validation**: It’s essential to evaluate the robustness of our findings across varied EEG datasets and populations. Such cross-dataset validation is crucial for real-world applications, necessitating further investigation.

### Conclusion

In conclusion, our findings strongly highlight the effectiveness of ROCKET-derived features, especially given the specialized design of the ROCKET algorithm for handling time series data intrinsic to EEG recordings. Its ability to discern patterns within the EEG data has proven to be a valuable asset in predicting secure and insecure attachment styles. On the other hand, the classic features also demonstrated a commendable capability in this prediction task. However, when choosing between these feature sets, researchers should take into account not only their respective strengths but also considerations like complexity, interpretability, and the overarching aim of achieving optimal predictive accuracy. The dynamic nature of EEG data might lean towards the use of ROCKET in many scenarios, but the classic features can offer interpretability and familiarity that some researchers may prioritize.

### Electronic supplementary material

Below is the link to the electronic supplementary material.

Supplementary Material 1

## Data Availability

All the experimental data, which includes the players’ electrophysiological recordings and the corresponding resource allocation logs, are stored on the servers of Ariel University. The data can be obtained by request from one of the authors.All the experimental data, which includes the players’ electrophysiological recordings and the corresponding coordination logs, are stored on the servers of Ariel University. The data can be obtained by request from The IRB member, Dr. Chen Hajaj (chenha@ariel.ac.il) or from one of the authors (Dor Mizrahi - dor.mizrahi1@msmail.ariel.ac.il, Ilan Laufer - ilanl@ariel.ac.il, Inon Zuckerman - inonzu@ariel.ac.il).

## References

[1] Bowlby J. Attachment and loss v. 3 (Vol. 1). Basic Books. 1969.

[2] Ainsworth MDS, Blehar MC, Waters E, Wall SN. Patterns of attachment: a psychological study of the strange situation. Psychology; 2015.

[3] Sloan EP, Maunder RG, Hunter JJ, Moldofsky H (2007). Insecure attachment is associated with the α-EEG anomaly during sleep. Biopsychosoc Med.

[4] Gander M, Buchheim A. Attachment classification, psychophysiology and frontal EEG asymmetry across the lifespan: a review. Front Hum Neurosci. 2015;9. 10.3389/fnhum.2015.00079 PMC4333768 25745393

[5] Cecchini M, Iannoni ME, Pandolfo AL, Aceto P, Lai C (2015). Attachment style dimensions are associated with brain activity in response to gaze interaction. Soc Neurosci.

[6] Dan O, Zreik G, Raz S (2020). The relationship between individuals with fearful-avoidant adult attachment orientation and early neural responses to emotional content: an event-related potentials (ERPs) study. Neuropsychology.

[7] Wang J, Wang M (2021). Review of the emotional feature extraction and classification using EEG signals. Cogn Robot.

[8] Lai C, Ciacchella C, Altavilla D, Veneziani G, Aceto P, Cecchini M et al. Attachment style dimensions are associated with neural activation during projection of mental states. Front Hum Neurosci. 2022;16. 10.3389/fnhum.2022.899418 PMC9387349 35992957

[9] Zhuang N, Zeng Y, Tong L, Zhang C, Zhang H, Yan B. Emotion recognition from EEG signals using multidimensional information in EMD domain. Biomed Res Int. 2017. 10.1155/2017/8317357 PMC5576397 28900626

[10] Chen T, Ju S, Ren F, Fan M, Gu Y. EEG emotion recognition model based on the LIBSVM classifier. Measurement. 2020;164.

[11] Rahman M, Sarkar AK, Hossain A, Moni MA. EEG-based emotion analysis using non-linear features and ensemble learning approaches. Expert Syst Appl. 2022;207.

[12] Jaswal RA, Dhingra S. Empirical analysis of multiple modalities for emotion recognition using convolutional neural network. Meas Sens. 2023;26.

[13] Vempati R, Sharma LD. A systematic review on Automated Human emotion recognition using Electroencephalogram Signals and Artificial Intelligence. Results Eng. 2023;101027.

[14] Gómez-Zaragozá L, Marín-Morales J, Vargas EP, Giglioli IAC, Raya MA. An online attachment style Recognition System based on Voice and Machine Learning. IEEE J Biomed Heal Inf. 2023. 10.1109/JBHI.2023.3304369 37566508

[15] Koçak TM, Dibek BÇ, Polat EN, Kafesçioğlu N, Demiroğlu C. Automatic detection of attachment style in married couples through conversation analysis. EURASIP J Audio, Speech, Music Process. 2023;1.

[16] Zong J, Xiong X, Zhou J, Ji Y, Zhou D, Zhang Q (2023). FCAN–XGBoost: a novel hybrid model for EEG emotion recognition. Sensors.

[17] Tiwari A, Chaturvedi A. A multiclass EEG signal classification model using spatial feature extraction and XGBoost algorithm. In: IEEE/RSJ International Conference on Intelligent Robots and Systems (IROS). 2019.

[18] Zuckerman I, Mizrahi D, Laufer I. Exploring EEG Features for Differentiating Between Secure and Insecure Attachment Styles. In: Proceedings of SAI Intelligent Systems Conference. Cham: Springer International Publishing; 2023.

[19] Laufer I, Mizrahi D, Zuckerman I. Enhancing EEG-Based attachment style prediction: unveiling the impact of feature domains. Front Psychol. 2024;15. 10.3389/fpsyg.2024.1326791 PMC10838989 38318079

[20] Mizrahi D, Laufer I, Zuckerman I (2023). The effect of feedback on Electrophysiological Signal Complexity as a function of attachment style. NeuroIS Retreat 2023.

[21] Mizrahi D, Laufer I, Zuckerman I. Modulation of Beta Power as a function of Attachment Style and Feedback Valence. In: International Conference on Brain Informatics. Hoboken, New jersey USA: Cham: Springer Nature Switzerland; 2023. p. 14–20.

[22] Dempster A, Petitjean F, Webb GI (2020). ROCKET: exceptionally fast and accurate time series classification using random convolutional kernels. Data Min Knowl Discov.

[23] Wong T-T (2015). Performance evaluation of classification algorithms by k-fold and leave-one-out cross validation. Pattern Recognit.

[24] Sun L. Application and improvement of xgboost algorithm based on multiple parameter optimization strategy. In: 2020 5th international conference on mechanical, control and computer engineering (icmcce). IEEE; 2020. p. 1822–5.

[25] Suresh GV, Reddy S (2022). Uncertain data analysis with regularized XGBoost. Webology.

[26] Adamczyk K, Pilarska A. Attachment style, relationship status, gender and relational competences among young adults. Pol Psychol Bull. 2012.

[27] Geirdal AØ, Nerdrum P, Bonsaksen T (2019). The transition from university to work: what happens to mental health? A longitudinal study. BMC Psychol.

[28] Different EEG (2020). Brain activity in right and left handers during visually induced self-motion perception. J Neurol.

[29] Sibley CG, Fischer R, Liu JH (2005). Reliability and validity of the revised experiences in close relationships (ECR-R) self-report measure of adult romantic attachment. Personal Soc Psychol Bull.

[30] Sibley CG, Liu JH (2004). Short-term temporal stability and factor structure of the revised experiences in close relationships (ECR-R) measure of adult attachment. Personal Individ Differ.

[31] Jain AK (2008). Data clustering: 50 years beyond K-means. Machine learning and knowledge Discovery in Databases.

[32] Brunetti M, Zappasodi F, Croce P, Di Matteo R (2019). Parsing the Flanker task to reveal behavioral and oscillatory correlates of unattended conflict interference. Sci Rep.

[33] Faouzi J. Time series classification: a review of algorithms and implementations. Mach Learn (Emerging Trends Appl; 2022.

[34] Ruiz AP, Flynn M, Large J, Middlehurst M, Bagnall A (2021). The great multivariate time series classification bake off: a review and experimental evaluation of recent algorithmic advances. Data Min Knowl Discov.

[35] Dempster A, Schmidt DF, Webb GI. Minirocket: A very fast (almost) deterministic transform for time series classification. In: Proceedings of the 27th ACM SIGKDD conference on knowledge discovery & data mining. 2021. p. 248–57.

[36] Tan CW, Dempster A, Bergmeir C, Webb GI (2022). MultiRocket: multiple pooling operators and transformations for fast and effective time series classification. Data Min Knowl Discov.

[37] Lundy C, O’Toole JM. Random convolution kernels with multi-scale decomposition for preterm EEG inter-burst detection. In: 29th European Signal Processing Conference (EUSIPCO). IEEE; 2021. p. 1182–6.

[38] Potter İY, Zerveas G, Eickhoff C, Duncan D. Unsupervised Multivariate Time-Series Transformers for Seizure Identification on EEG. In: 21st IEEE International Conference on Machine Learning and Applications (ICMLA). IEEE; 2022. p. 1304–11.

[39] Dhariyal B, Nguyen T, Le, Ifrim G. Back to basics: a sanity check on Modern Time Series classification algorithms. arXiv Prepr. 2023;arXiv:2308.

[40] Duru AD (2019). Determination of increased mental workload condition from EEG by the use of classification techniques. Int J Adv Eng Pure Sci.

[41] Liu Z, Song J. Comparison of Tree-based Feature Selection Algorithms on Biological Omics Dataset. In: Proceedings of the 5th International Conference on Advances in Artificial Intelligence. 2021.

[42] Awotunde JB, Folorunso SO, Imoize AL, Odunuga JO, Lee C-C, Li C-T (2023). An ensemble tree-based model for intrusion detection in Industrial Internet of things networks. Appl Sci.

[43] Chen W, Cai Y, Li A, Su Y, Jiang K (2023). EEG feature selection method based on maximum information coefficient and quantum particle swarm. Sci Rep.

