# Supplementary material for: Comparative analysis of ROCKET-driven and classic EEG features in predicting attachment styles
Source: BMC Psychol. 2024 Feb 22;12:87. doi: 10.1186/s40359-024-01576-1 (PMC10882770; doi:10.1186/s40359-024-01576-1)
Supplement: Supplementary file 1 — Supplementary Material 1 [file 40359_2024_1576_MOESM1_ESM.docx]

**Appendix**

**Appendix A: Classification of Classic EEG-Based Features**

The Appendix A1, titled "The 45 Classic EEG-Based Features Used in the Study," provides a detailed overview of the conventional EEG features employed in our research. These features, essential to our analysis, are categorized into four distinct groups: Time-Domain Features, Complexity Features, Frequency-Based Features, and Trial-Feedback Dynamics. Each category encompasses a range of specific attributes, meticulously chosen to capture the intricate dynamics of EEG signals in the context of attachment styles. This comprehensive compilation serves as a foundational reference for understanding the EEG analysis performed in our study.

**Table A1: The 45 Classic EEG-Based Features Used in the Study**

| **Time-Domain Features** | **Complexity Features** | **Frequency-Based Features** | **Trial-Feedback Dynamics** |
| --- | --- | --- | --- |
| - Mean Absolute Energy - Max Amplitude - Sum of Absolute Changes - Count above Mean - Count above Median - First Max Location - First Min Location - Kurtosis - Last Max Location - Last Min Location - Longest Strike Above Mean - Longest Strike Above Median - Mean Absolute Change - Mean Change - Number of Crossing Mean - Number of Crossing Median - Range Count 25% to 75% - Skewness - Variation Coefficient | - Binned Entropy (2,4,8,16,32) - Fourier Entropy (2,4,8,16,32) - LZC (2,4,8,16,32) - cid_ce - Sample Entropy | - Relative Delta Power - Relative Theta Power - Relative Alpha Power - Relative Beta Power - Theta to Alpha Ratio (TAR) - Theta to Beta Ratio (TBR) | - Response Time (ms) - Feedback Valence (Current Trial) - Feedback Valence (Previous Trial) |

For detailed definitions of the time-domain and complexity EEG features used in our study, such as 'count above mean/median', 'first max location', 'min location', ‘sample entropy’ etc., please refer to the tsfresh documentation, available at <https://tsfresh.readthedocs.io/en/latest/text/list_of_features.html>. For the extraction of frequency-based features we used Discrete Wavelet Transform (DWT) [44, 45] as detailed in [The Effect of Individual Coordination Ability on Cognitive-Load in Tacit Coordination Games].
